# Biologically Active Metabolites Produced by the Basidiomycete *Quambalaria cyanescens*

**DOI:** 10.1371/journal.pone.0118913

**Published:** 2015-02-27

**Authors:** Eva Stodůlková, Ivana Císařová, Miroslav Kolařík, Milada Chudíčková, Petr Novák, Petr Man, Marek Kuzma, Barbora Pavlů, Jan Černý, Miroslav Flieger

**Affiliations:** 1 Institute of Microbiology of the ASCR, v.v.i., Prague, Czech Republic; 2 Department of Inorganic Chemistry, Faculty of Science, Charles University, Prague, Czech Republic; 3 Department of Cell Biology, Faculty of Science, Charles University, Praha, Czech Republic; Umeå Plant Science Centre, Umeå University, SWEDEN

## Abstract

Four strains of the fungus *Quambalaria cyanescens* (Basidiomycota: Microstromatales), were used for the determination of secondary metabolites production and their antimicrobial and biological activities. A new naphthoquinone named quambalarine A, (S)-(+)-3-(5-ethyl-tetrahydrofuran-2-yliden)-5,7,8-trihydroxy-2-oxo-1,4-naphthoquinone (**1**), together with two known naphthoquinones, 3-hexanoyl-2,5,7,8-tetrahydroxy-1,4-naphthoquinone (named here as quambalarine B, **2**) and mompain, 2,5,7,8-tetrahydroxy-1,4-naphthoquinone (**3**) were isolated. Their structures were determined by single-crystal X-ray diffraction crystallography, NMR and MS spectrometry. Quambalarine A (**1**) had a broad antifungal and antibacterial activity and is able inhibit growth of human pathogenic fungus *Aspergillus fumigatus* and fungi co-occurring with *Q. cyanescens* in bark beetle galleries including insect pathogenic species *Beauveria bassiana*. Quambalarine B (**2**) was active against several fungi and mompain mainly against bacteria. The biological activity against human-derived cell lines was selective towards mitochondria (**2** and **3)**; after long-term incubation with **2**, mitochondria were undetectable using a mitochondrial probe. A similar effect on mitochondria was observed also for environmental competitors of *Q. cyanescens* from the genus *Geosmithia*.

## Introduction

Fungi are a rich source of secondary metabolites, including valuable biologically active products with broad applications for humans. Intensively studied are saprotrophic and easily cultivable fungi of the phylum Ascomycota or particular groups of the Muccoromycota and Basidiomycota. By contrast, parasitic and symbiotic lineages have remained neglected. One such group is a diverse class of true smut fungi, Ustilaginomycetes (Basidiomycota). This group comprises parasites and saprotrophs restricted mainly to angiosperms [1]. *Quambalaria* is a genus of asexual fungi belonging to the order Microstromatales (Ustilaginomycetes: Exobasidiomycetidae). Five species of *Quambalaria* (excluding the dubious *Q. pussila*) have been described as parasites of *Eucalyptus* and other native Australian trees, being also introduced to other continents and hosts [2,3,4,5]. *Quambalaria cyanescens* (syn. *Sporothrix cyanescens*) is unique in its broad ecological niche. This fungus is associated with cankers on *Eucalyptus* and *Corymbia*, but is not pathogenic. It is regarded as an opportunistic pathogen in compromised human hosts and was also isolated several times from the air, insect larvae, soil and various plant materials worldwide [2,3,6,7,8]. Finally, it is commonly associated with bark beetles feeding on various plant hosts ([9], Kolařík, unpublished data). This hyaline filamentous fungus produces masses of blue to violet pigment that diffuses into cultivation media or the phloem in galleries of bark beetles. Noticeable is its competitive ability, which often results in the formation of monocultures inside bark beetle gallery systems. This species is a producer of the antibiotic sesquiterpene globulol [10]. The strong activity of its crude extract against various fungi and bacteria has already been documented in two undetermined *Quambalaria* species, both producing distinct reddish to violet pigment and being relatives of *Q. cyanescens* [11,12].

Naphthoquinones are widespread in nature and have often been found in higher plants, fungi and some bacteria [13,14,15,16,17,18]. Polyhydroxylated 1,4-naphthoquinones have also been isolated from sea urchins [19]. They are produced in large structural variety exhibiting diverse activities, for example, antimicrobial, antiviral, antifungal, anti-inflammatory, antimalarial, mutagenic, phytotoxic, insecticidal and recently extensively studied anticancer effects [20,21,22,23]. Another important feature of naturally produced naphthoquinones is their biotechnological potential. Many of them are known as colourants for cosmetics, fabrics and food.

Here we report the results of submerged culture production, isolation and structure determination of naphthoquinone derivatives produced by *Q. cyanescens* together with an evaluation of their antimicrobial and biological activity. We identified one new and two already known naphthoquinones, previously found in poorly documented fungal species. Two of them have antifungal properties and exhibit activity against mitochondria in human-derived carcinoma cell lineages. This research demonstrates that bright colored pigments produced by little explored smut fungi have potential in antimicrobial and anticancer drug research.

## Experimental procedures

Strain isolation and identification. All experiments were done on the strain *Quambalaria cyanescens* CCM 8372 ( = MK1710), isolated from *Scolytus intricatus*, Bulgaria, Bachkovo, 41° 56′ 28.11″ N, 24° 50′ 53.69″ E, izol. M. Kolařík, May 2005. Three other *Q. cyanescens* strains were used for comparison: CCF 3528 isolated from *Phloetribus scarabeoides*, Croatia, Brač island, 43° 17′ 6″ N, 16° 52′ 32″ E, izol. M. Kolařík, Aug. 2004; MK755 isolated from *Scolytus amygdali*, Syria, Baniyas, Al Marquab, 35° 8′ 60″ N, 35° 57′ 0″ E, izol. M. Kolařík, Apr. 2004; and CCM 8373 isolated from bark beetle feeding on *Arbutus unedo*, Tunnisia, Sousse, 35° 53′ 15.5″ N 10° 35′ 58.8″ E. No specific permissions were required for the location/activity and the field studies did not involve endangered or protected species. The strains were identified using ITS rDNA sequences (deposited under the Genbank accession no. AM261922.2, AM 262921, and DQ119134), morphology and physiological traits [9]. Strains are deposited in the Czech collection of microorganisms (CCM, Brno, Czech Republic.) and in the Culture collection of fungi (CCF, Prague, Czech Republic).

Cultivation conditions. The stock culture of the monosporic strain *Q. cyanescens* CCM 8372 was maintained on malt agar slants (malt extract 20 g/L, agar 20 g/L) and cultivated on a Czapek-Dox liquid medium containing (g/L): sucrose 30, agar 20, NaNO_3_ 3, K_2_HPO_4_ 1, MgSO_4_ 0.5, KCl 0.5, Fe_2_(SO_4_)_3_ 0.01; pH 6.5. Submerged cultivations were carried out in 250 mL Erlenmeyer flasks on a rotary shaker (200 rpm) for 21 days at 24°C in the dark.

Pigment extraction. The fermentation broth of *Q. cyanescens* CCM 8372 (7 L) was centrifuged, filtered and extracted with an equal volume of ethyl acetate containing 3% (V/V) of acetic acid (3 times). Pooled extracts were dried over anhydrous Na_2_SO_4_, filtered and evaporated to dryness under reduced pressure to produce a dark violet solid (3.2 g).

Column chromatography. The crude extract (700 mg) diluted in CH_2_Cl_2_ was subjected to gel chromatography on Sephadex LH-20 (150 g, GE Healthcare Bio-Science, Sweden) equilibrated in the same solvent and eluted with CH_2_Cl_2_ followed by a stepwise gradient of MeOH in CH_2_Cl_2_ (0.5: 8; 1: 8; 1:1 (V/V), and finally with pure MeOH. Fractions were collected and combined based on their TLC profiles (toluene/ethyl acetate/TFA; 6:4:1; v/v/v). The compounds eluted in the following sequence: **1**, **2** and **3**. Fractions containing pure compounds were combined and repeatedly crystallized from a solvent mixture of CH_2_Cl_2_/MeOH. The fraction containing compound **1** yielded brown-cinnamon crystals (170 mg), metabolite **2** was obtained as deep violet crystals (50 mg), and the known naphthoquinone mompain (**3**) formed red crystals (80 mg).

Mass spectrometry. Mass spectrometric experiments were performed using a commercial 9.4T APEX-Ultra FTMS instrument (Bruker Daltonics, Billerica MA) equipped with an ESI/MALDI ion source. The analysis was performed using an electrospray ionization (ESI) and the spectra were acquired in positive and negative ion mode. The cell was opened for 1.3 msec, accumulation time was set at 0.2 s for MS experiment (1.0 s for MS/MS experiment), and one experiment was collected for each sample where one experiment consists of the average of eight spectra. After MS experiment one MS/MS experiments was done from the ion of interest (S1 Fig.). The isolation window was set 4 a.m.u. and the collision energy was kept at 16V (negative mode). The acquisition data set size was set to 1M points with the mass range starting at m/z 150 a.m.u., resulting in a resolution of 100,000 at m/z 400. One μl of each sample was dissolved in 0.1 ml of MeOH and introduced into the mass spectrometer by direct infusion into the electrospray ion source. The instrument was externally calibrated using singly charged arginine clusters, resulting in sub-ppm accuracy. The spectra were apodized using sine apodization with one zero filling. Data were processed in the software Data Analysis 4.0, and the possible elemental compositions were calculated using Smart Formula calculations.

NMR spectrometry. NMR spectra were recorded on a Bruker Avance III 600 MHz spectrometer (600.23 MHz for ^1^H, 150.93 MHz for ^13^C at 30°C) in DMSO-d6 (99.8 atom% D, ARMARChemicals, Dottingen, Switzerland). Residual signals of solvents were used as an internal standard (δH 2.500 ppm, δC 39.60 ppm). NMR experiments: ^1^H NMR, ^13^C NMR, COSY, ^1^H-^13^C HSQC, and ^1^H-^13^C HMBC were performed using the manufacturer’s software. The ^1^H and ^13^C NMR spectra were zero filled to fourfold data points and multiplied by window function before Fourier transformation. Two-parameter double-exponential Lorentz-Gauss function was applied for ^1^H to improve resolution and line broadening (1 Hz) was applied to get better ^13^C signal-to-noise ratio. Chemical shifts are given in δ-scale with digital resolution justifying the reported values to three (δH) or two (δC) decimal places. ^1^H NMR, ^13^C NMR data of the compound **1** and **2** are presented in S2 Fig.

Compound **1** (quambalarine A): ^1^H NMR (600.23 MHz, DMSO-*d*_*6*_, 303.1 K): δ 13.885 (s), 13.697 (s), 13.208 (s), 13.058 (s), 12.199 (s), 12.178 (s), 11.127 (s), 11.093 (s), 6.683 (s, = CH-), 6.669 (s, = CH-), 6.331 (s, = CH-), 5.007 (m, CH), 4.981 (m, CH), 4.400 (m, CH), 3.691 (ddd, J = 4.2, 9.4, 20.2 Hz), 3.598 (ddd, J = 4.2, 9.4, 20.2 Hz), 3.480 (ddd, J = 8.3, 9.7, 20.2 Hz), 3.409 (ddd, J = 8.2, 9.6, 20.2 Hz), 2.512 (td, J = 8.8, 17.6 Hz), 2.442 (ddd, J = 4.4, 9.4, 17.6 Hz), 2.305 (m), 2.234 (dddd, J = 4.3, 6.7, 9.6, 12.5 Hz), 1.843–1.724 (m), 1.633 (m), 1.572 (m), 1.013 (t, J = 7.4 Hz), 1.004 (t, J = 7.4 Hz), 0.897 (t, J = 7.4 Hz).

^13^C NMR (150.93 MHz, DMSO-*d*_*6*_, 303.1 K): δ 193.48 (s), 192.80 (s), 187.42 (s), 185.11 (s), 183.29 (s), 183.22 (s), 178.10 (s), 177.21 (s), 174.89 (s), 172.78 (s), 166.74 (s), 158.27 (s), 158.02 (s), 157.57 (s), 154.05 (s), 153.78 (s), 148.46 (s), 148.30 (s), 113.79 (s), 113.78 (s), 111.82 (d), 111.80 (d), 111.56 (d), 111.03 (d), 110.84 (d), 110.64 (d), 105.63 (s), 105.29 (s), 103.31 (s), 92.78 (d), 92.30 (d), 81.56 (d), 38.36 (t), 38.10 (t), 28.46 (t), 27.87 (t), 27.37 (t), 27.31 (t), 26.93 (t), 26.09 (t), 25.83 (t), 9.68 (q), 9.66 (q), 9.43 (q).

Compound **2** (quambalarine B): ^1^H NMR (600.23 MHz, DMSO-*d*_*6*_, 303.1 K): δ 6.537 (1H, s, H-2), 2.792 (1H, t, J = 7.3 Hz, H-12), 1.578 (1H, m, H-13), 1.293 (4H, m, H-14, H-15), 0.866 (1H, t, J = 7.1 Hz, H-16).

^13^C NMR (150.93 MHz, DMSO-*d*_*6*_, 303.1 K): δ 202.63 (s, C-11), 177.79 (s, C = O), 175.14 (s, C = O), 165.43 (s, = C-O), 158.13 (s, = C-O), 156.44 (s, = C-O), 121.53 (s, = C-), 111.84 (s, = C-), 111.04 (d, = CH-), 103.00 (s, = C-), 43.22 (t, C-12), 30.73 (t, C-14), 22.70 (t, C-13), 21.90 (t, C-15), 13.80 (q, C-16).

X-ray crystallography. Single-crystal X-ray diffraction data for compounds **1**–**3** were obtained with a Nonius KappaCCD diffractometer using monochromatized MoKα radiation (λ = 0.71073 Å) at 150(2)K. The structures were solved by direct methods [24] and refined by a full-matrix least squares treatment based on *F* ^2^ [25]. Hydrogen atoms on carbons were fixed into idealized positions (riding model) and assigned temperature factors of either *U*_iso_(H) = 1.2 *U*_eq_(pivot atom) or 1.5 U_eq_ for methyl moiety. The hydrogen atoms of hydroxyls were located from the difference Fourier map and refined as riding atoms with *U*_iso_ fixed at 1.2 *U*_eq_ of the parent oxygen atoms.

Crystal data of the compound **1**. Crystal formula, C_16_H_14_O_7_·CH_2_Cl_2_·CH_4_O, triclinic, space group *P-*1 (No 2); *a* = 7.9667 (5) Å, *b* = 9.7312 (7) Å, *c* = 13.7074 (9) Å, *α* = 70.887 (4)°, *β* = 80.739 (4)°, *γ* = 70.215 (3)°; *V* = 943.32 (11) Å^3^; Z = 2; *D*_x_ = 1.532 Mgm^-3^; *μ* = 0.39 mm^-1^ dimensions of crystal 0.2 × 0.15 × 0.05 mm; 15421 diffractions, 3354 independent (*R*_int_ = 0.049), 266 parameters, *R*[*F* ^2^ > 2σ(*F* ^2^)] = 0.065, *w*R(*F* ^2^) = 0.145, min/max residual electron density −0.30/0.27 eÅ^-3^. The ethyl moiety as well as its pivot atom in pyrano-ring are disordered in two positions with the ration 0.6:0.4.

Crystal data of the compound **2**. Crystal formula C_16_H_14_O_7_, triclinic, space group *P-*1 (No 2); *a* = 4.8675 (2) Å, *b* = 8.9046 (4) Å, *c* = 16.0081 (9) Å, *α* = 91.968 (3)°, *β* = 91.039 (3)°, *γ* = 94.127 (3)°; *V* = 691.49 (6) Å^3^; Z = 2; *D*_x_ = 1.529 Mgm^-3^; *μ* = 0.12 mm^-1^ dimensions of red crystal 0.5 × 0.15 × 0.05 mm; 10618 diffractions, 2185 independent (*R*_int_ = 0.078), 209 parameters, *R*[*F* ^2^ > 2σ(*F* ^2^)] = 0.081, *w*R(*F* ^2^) = 0.246, min/max residual electron density-0.44/ 0.43 eÅ^-3^.

Crystal data of the compound **3**. Crystal formula C_10_H_6_O_6_•CH_4_O, triclinic, space group *P-*1 (No 2); *a* = 6.5764 (10) Å, *b* = 8.6518 (9) Å, *c* = 9.5632 (13) Å, *α* = 97.611 (8)°, *β* = 103.229 (7)°, *γ* = 99.834 (8)°; *V* = 513.49 (12) Å^3^; Z = 2; *D*_x_ = 1.644 Mgm^-3^; *μ* = 0.14 mm^-1^ dimensions of pink crystal 0.50 × 0.12 × 0.02 mm; 7781 diffractions, 1821 independent (*R*_int_ = 0.053), 164 parameters, *R*[*F* ^2^ > 2σ(*F* ^2^)] = 0.056, *w*R(*F* ^2^) = 0.168, min/max residual electron density-0.28/ 0.22 eÅ^-3^.

Antimicrobial and antifungal activity. The antibiotic activity of the crude extract of *Q. cyanescens* CCM 8372 and isolated compounds **1–3** was tested in vitro against the following bacteria: *Kocuria rhizophila* CCM 552 ( = ATCC 9341, previously known as *Micrococcus luteus*), *Escherichia coli* ATCC 3988; yeasts: *Saccharomyces cerevisiae* CCM 8191 ( = ATCC9763), *Candida albicans* CCM 8215; filamentous fungus *Aspergillus fumigatus* CEA10 and fungi occurring in bark beetle galleries: *Geosmithia* sp. 9 strain RJ0258, *Geosmithia* sp. 2 CCF 4273 [26] and *G. langdonii* CCF 3332 [27], the ophiotomatoid fungus and endophyte *Graphium fimbriisporum* CCF 4421, the wood saprophyte *Penicillium decumbens* CCF 4423 and the insect pathogenic species *Beauveria bassiana* CCF 4422 [28]. Bacteria were cultivated on a beef extract medium containing (g/L): beef extract 10, peptone 10, NaCl 5 and agar 20; pH 7.2 adjusted by NaOH. The yeasts were cultivated on a yeast extract medium consisting of (g/L): glucose 40, peptone 5, yeast extract 5 and agar 20; pH 7.0 adjusted by NaOH. Filamentous fungi were cultivated on a MEA medium containing (g/L): malt extract 20, glucose 20, peptone 1 and agar 20. The suspension of each microbial strain was inoculated on the surface of the appropriate medium in a Petri dish. The crude extract and individual substances (20 μl of stock solution, 1 mg/ml in MeOH) were subsequently loaded into the wells made with a cork borer (6–8 on each dish). A well containing only MeOH was assayed as a blank control and did not inhibit the growth of any of the microbial strains. Antimicrobial activity was determined as the semi diameter (mm) of the observed growth inhibition zone. Commercially available antibiotics cycloheximide, chloramphenicol, streptomycine, antimycine A (all from Sigma-Aldrich) were used for comparison.

Detailed analysis of antifungal activity against *Geosmithia* sp. Small pieces of mycelia from *Geosmithia* sp. 9 RJ0258 and *Q. cyanescens* CCM 8372 (four from each fungus, approx. 2×2 mm) were excised and incubated in 100 μl of PBS with or without 20 μM of **2** followed by 10 min co-incubation with MitoTracker Red CMXRos and cell CellROX Green Reagent. Mycelia were mounted between a glass slide and a coverslip, gently squeezed and imaged *in vivo* using a Cell (Olympus) inverted microscopic system optimized for live cell imaging using a 63× immersion objective. For each sample, 8–12 layers in the Z direction were acquired (spaced 500 nm), processed using deblurring algorithms and overlaid using the maximum projection intensity Z algorithm, which provides information about the distribution of fluorescent signals in the approx. 5 μm thickness of the specimen.

Biological activity. HeLa, HEK 293, HTC 116 and A549 cancer-derived cell lines (only representative data from HeLa cell line are included in the manuscript) were cultivated in a D-MEM medium supplemented with 10% FCS (Gibco, Invitrogen, Carlsbad, CA, USA) and grown on glass cover slips (up to 50% density) in 6-well plates (Nunc, Thermo Fisher Scientific, Waltham, MA, USA) treated with the compounds dissolved at various concentrations in DMSO (stock solution 10 mM) for different times. Wells containing DMSO only (max. 1%) were used as blank controls—no alteration of cellular morphology and physiology was observed. Treated cells (various times and concentrations) grown at 37°C in a humidified 5% CO_2_ atmosphere were treated with fluorescent trackers (Lysotracker Red, Holotransferrin Alexa Fluor 594 conjugate or MitoTracker Red CMXRos) and visualized *in vivo* or fixed (3.7% paraformaldehyde in PBS, 20 min, RT), permeabilized (0.1% Triton X-100 in PBS), blocked (1% BSA in PBS) and stained with Phalloidin-Alexa Fluor 488 conjugate, anti-tubulin antibody TU-1 (EXBIO, Vestec, Czech R.) or antibody against LAMP-2 protein MEM 259 (EXBIO, Vestec, Czech R.). Staining of nuclei was performed by mounting specimens in Mowiol-DAPI or *in vivo* in cells with permeable plasma membranes by diethidium bromide. All fluorescent reagents were purchased from Molecular Probes (Invitrogen, Carlsbad, CA, USA). Visualization of all specimens was performed using a Cell microscope (Olympus) using a 63× immersion objective. Approximately 20 layers in the Z direction were acquired (spaced 250nm) and processed by the same procedure as mycelium of *Geosmithia* sp. All experiments were performed in triplicates and repeated at least two times with reproducible results.

Additional suspension tumor lines used for flow cytometry experiments were of hematopoietic origin (REH, NALM 6 and Jurkat). Representative example of the FACS-based bioassay of the mitochondrial activity performed on Jurkat cell line is presented in S1 File.

## Results and Discussion

**Fig 1:**
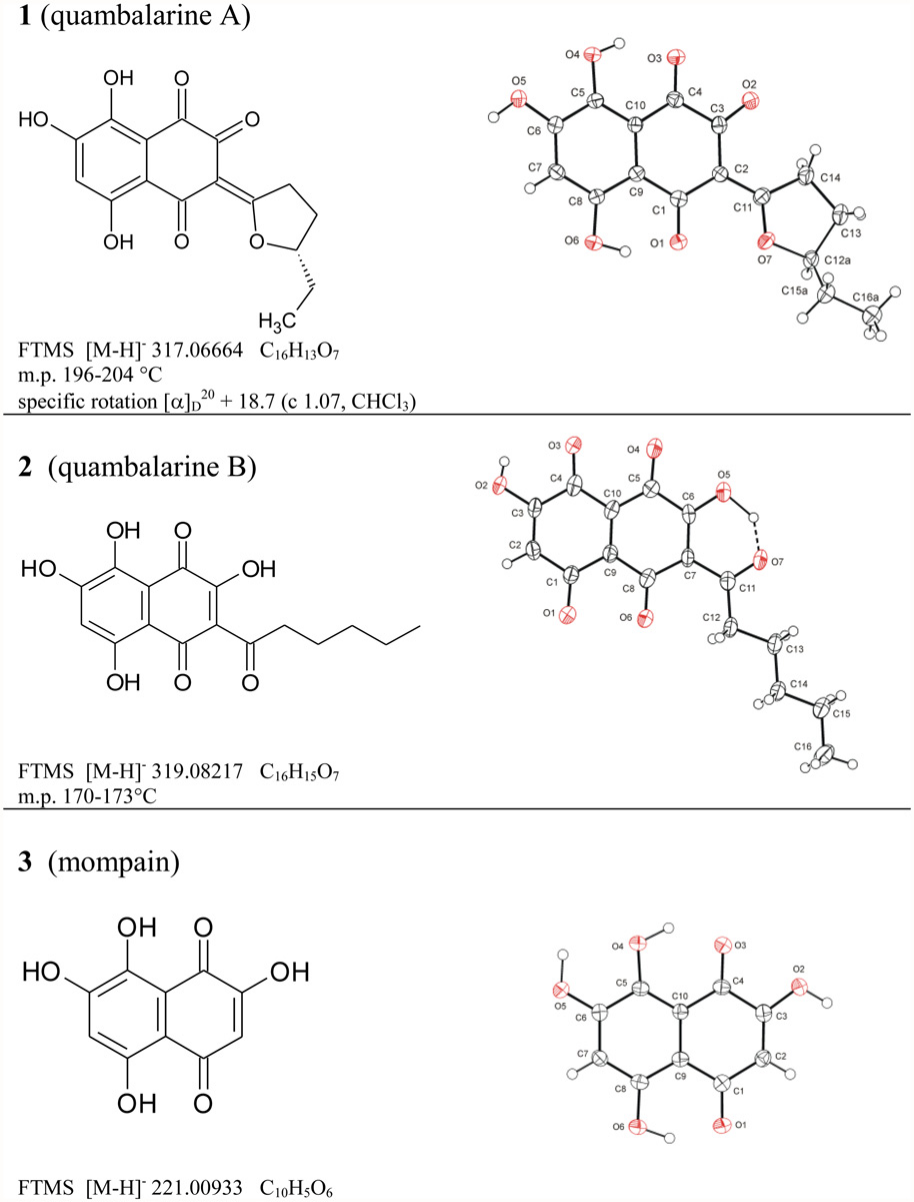
Left column: Structural formulas, FTMS data, and physical characteristics of naphthoquinones isolated from submerged culture of *Quambalaria cyanescens*; right column: X-ray structures, the displacement ellipsoids are drawn on 50% probability level.

Gel chromatography of the crude extract of the fermentation broth of *Q. cyanescens* CCM 8372 led to the isolation of three naphthoquinone-derived compounds. All isolates crystallized well from the mixture of CH_2_Cl_2_/MeOH, so their structures were determined directly by single crystal X-ray analysis. The molecular formulas determined by HRMS satisfactorily agreed with X-ray crystallographic (Fig. 1) and NMR data. The determination of the absolute configuration of compound **1**was based on measured specific rotation ([α]_D_^20^ + 18.7 (c 1.07, CHCl_3_)) and its close structure similarity with published data on γ-methyl-γ-butyrolactone [29]. Therefore compound **1** is a new naphthoquinone, (S)-(+)-3-(5-ethyl-tetrahydrofuran-2-yliden)-5,7,8-trihydroxy-2-oxo-1,4-naphthoquinone while compound **2** (3-hexanoyl-2,5,7,8-tetrahydroxy-1,4-naphthoquinone) and mompain **3** (2,5,7,8-tetrahydroxy-1,4-naphthoquinone) were described earlier from an unspecified yeast and the plant parasitic basidiomycete *Helicobasidium mompa*, respectively [30,31]. The new compound **1** is named here as quambalarine A and compound **2** as quambalarine B. Pure compounds **1–3** together with the crude extract were further used for the testing of their antimicrobial, antifungal and biological activities.

Gel chromatography analysis of the crude extract showed presence of the same set of pigments in other three tested *Q. cyanescens* strains. Production of the bluish pigment is species specific character of *Q. cyanescens* [7] and the same compounds and their derivates can be expected across the species. Other potential producers of naphthoquinones are related smut fungi, such as *Exobasidium vexans*, *E. japonicum*, *Tilletiopsis minor*, *T. fulvescens* [32] *Meira geulakonigii* [33], *M. nashicola* [34], which produce reddish coloured and often antibiotic pigments in culture.

Biological activity. Microscopy was used to characterize cellular morphology, viability, cytoskeleton organization, integrity and dynamics of vesicular structures to study the biological activity of isolated naphthoquinones on various tumour cell lines i.e., HeLa, HEK 293, HTC 116 and A549. Flow cytometry was used to study REH, NALM 6 and Jurkat cell lines.

Cells were incubated (30 min—24 h) with naphthoquinones in the concentration range 0–250 μM. Different bioactivities were observed when structurally similar compounds were applied. None of the secondary metabolites tested (25 μM) was cytotoxic in incubations shorter than 10 h. Only extremely high concentrations (250 μM) lead in a matter of hours to cell death. HeLa cells treated with physiologically relevant concentrations (25 μM) of the tested compounds maintained their adhesion and characteristic morphology, the functionality of endosomes/lysosomes, an intact structure of the cytoskeleton (actin and tubulin), and the intactness of the cell nucleus. Quambalarine B (**2)** and mompain (**3)** but not quambalarine A (**1)** slowed down the cell division in a concentration-dependent manner.

**Fig 2:**
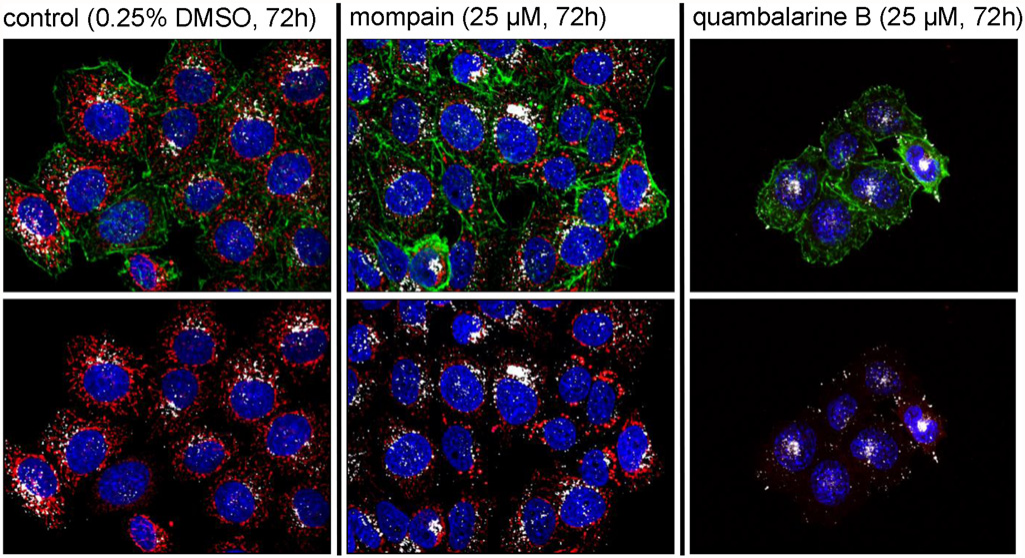
Effect of quambalarine B (2) and mompain (3) on the adenocarcinoma cell line HeLa. 0.25% DMSO was used as a solvent and as a control. Upper panels: visualization of mitochondria using MitoTracker Red CMXRos (red), actin cytoskeleton using Phalloidin (green), lysosomes using monoclonal antibody MEM 259 recognizing the lysosomal protein LAMP2 (white) and nuclei with DAPI (blue). Lower panels: simultaneous visualization of mitochondria, lysosomes and nuclei only.

Noticeable was the selectivity of the effect for only one type of organelle, mitochondria, which could be explained by the fact that mitochondria differ from the rest of the cell due to their evolutionary origin through endosymbiosis. A typical result of such phenotypic visualization is shown in Fig. 2. The effect of mompain (**3**) on the mitochondrial system consists in transforming mitochondrial networks into vesicular formations resembling the arrangement of mitochondria during cell division while complete disappearance of mitochondria was observed when cells were treated with quambalarine B (**2**). A clear fluorescence signal of the MitoTracker Red CMXRos probe indicating charging of the proton gradient observed at the beginning of treatment with **2** disappeared within 48 h and the cells remained viable at least for the next five days. For the whole period cells were able to endocytose fluorescently labelled transferrin and had membranes impermeable to DAPI or diethidium bromide.

Quantitation of the drop in the MitoTracker Red CMXRos signal after incubation with quambalarine B (**2**) was performed using flow cytometry. Cancer-derived cell lines of the haematopoietic origin (REH, NALM 6 and Jurkat) were treated with various concentrations (5–30 μM) of quambalarine B (**2**). Treatment of the cells resulted in the increase (concentration dependent manner) of the portion of MitoTracker Red CMXRos negative cells (see S1 File).

Antimicrobial activity. To test the general antimicrobial activity of the crude extract and compounds **1–3**, representatives of gram positive, gram negative bacteria, yeast and filamentous fungi were selected. Because of the observed antifungal activity of *Q. cyanescens*, some ecologically competing fungi, namely *Geosmithia*, *Graphium* and *Beauveria*, were also selected. The results are summarized in Table 1 and S3 Fig. Crude extract and quambalarine A (**1**) had broad antifungal and antibacterial activity and inhibited growth of human pathogenic fungus *A. fumigatus*. Quambalarine B (**2**) was active against several fungi and mompain (**3**) mainly against bacteria. Inhibitory activity of the crude extract on the growth of various fungi and bacteria were already reported in fungal strains related to or identical with *Q. cyanescens* [12] or strain related to *Q. eucalypti* [11]. Our results show that *Q. cyanescens* has a species-specific antibiotic effect and can inhibit the growth of fungi associated with bark beetles, including insect pathogenic fungi.

**Table 1:** Antibacterial and antifungal activity of naphthoquinones isolated from submerged culture of *Quambalaria cyanescens* and their comparison with commercial antibiotics.

Strain	compound							
	C.E.	1	2	3	CH	C	S	A
*Kocuria rhizophila*	3	2	n.o.	4	11	n.o.	9	n.o.
*Escherichia coli*	1	2–3	n.o.	3	11	n.o.	7	n.o.
*Saccharomyces cerevisiae*	2	6	1	1	n.o.	14	n.o.	10*
*Candida albicans* CCM 8215	1	n.o.	n.o.	n.o.	n.o.	n.o.	n.o.	12
*Geosmithia* sp. 9 RJ0258	5	6	2	n.o.	n.o.	27	n.o.	5
*Geosmithia* sp. 2 CCF4273	1	4	1	n.o.	n.o.	5	n.o.	6
*G. langdonii* CCF3332	2	3	1	n.o.	n.o.	5	n.o.	5
*Graphium fimbriisporum* CCF4421	3	5	1	n.o.	n.o.	15	n.o.	2
*Penicillium decumbens* CCF4423	n.o.	n.o.	n.o.	n.o.	n.o.	3	n.o.	2
*Beauveria bassiana* CCF4422	2	4	n.o.	n.o.	n.o.	n.o.	n.o.	n.o.
*Aspergillus fumigatus* CEA10	1	3	n.o.	n.o.	n.o.	2	n.o.	n.o.

20 μl (1 mg/mL) was loaded in all cases. Activities are expressed as a semi diameter of the observed growth inhibition zone (mm).

C.E. crude extract from the strain *Q. cyanescens* CCM 8372; CH chloramphenicol, C cycloheximide, S Streptomycine, A Antimycine A; n.o. not observed

**Fig 3:**
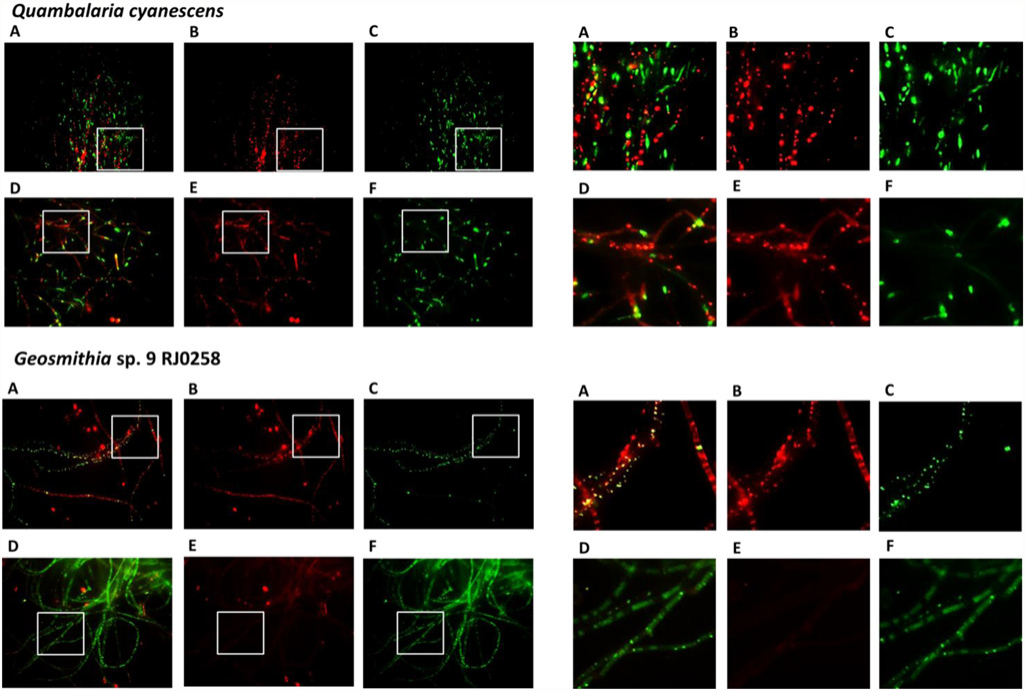
Effect of quambalarine B (2) on *Quambalaria cyanescens* and *Geosmithia sp*. *9* RJ0258. Both fungal species were treated for 2h with 20 μM quambalarine B, active mitochondria were detected using MitoTracker Red CMXRos (panels B and E), ROS were detected using CellROX Green Reagent (C and F), panels A and D shows overlay of mitochondrial and ROS signals. Upper array of images shows control cells, lower one cells treated with quambalarine B. Magnified representative regions are shown in right panels.

To explain the antifungal activity of quambalarine B (**2**) against *Geosmithia* sp. 9 RJ0258, we focused on mitochondrial morphology observed in human-derived cancer cells as a potential target and on the production of reactive oxygen species (ROS) as a potential molecular mechanism of cytotoxicity. Incubation (2 h) of *Geosmithia* sp. 9 RJ0258 with quambalarine B (**2**, 20 μM, Fig. 3) led to significant increase in the production of ROS and was accompanied by a drop in the mitochondrial proton gradient. This indicates that the mitochondrial dysfunction could be a potential mechanism of the compound cytotoxicity. No such effects were observed when *Q. cyanescens*, the producer of compound **2**, was studied (Fig. 3).

The antimicrobial and biological activities described above are extremely interesting due to their selectivity for only one organelle type which is the centre of the cellular energetic metabolism and regulation of apoptosis. In this context, it is important to mention that the tumour and normal cells often differ in the functioning of the mitochondrial system (e.g. Wartburg effect), and therefore a selective inhibitor could affect cancer cells differently. Another possible use of quambalarine B (**2)** is to influence the ratio of pathological to normally functioning populations of mitochondria in patients with mitochondrial hereditary diseases. It can be assumed that pathological mitochondria with a mutated mitochondrial genome (and therefore altered functionality) can be affected by quambalarine B (**2)** and mompain (**3)** differently from physiological ones.

## Supporting Information

S1 Fig Collision-induced dissociation (CID) data from MS. (DOCX)

S2 Fig Copies of 1H NMR and 13C NMR spectra. (PDF)

S3 Fig Antibacterial and antifungal activity of compounds tested in Table 1 (photodocumentation). (DOCX)

S1 File Effect of quambalarine B on Jurkat cell line. (DOCX)
