# Supplementary material for: Biologically Active Metabolites Produced by the Basidiomycete Quambalaria cyanescens
Source: PLoS One. 2015 Feb 27;10(2):e0118913. doi: 10.1371/journal.pone.0118913 (PMC4344228; doi:10.1371/journal.pone.0118913)
Supplement: S1 Fig — (DOCX) [file pone.0118913.s001.docx]

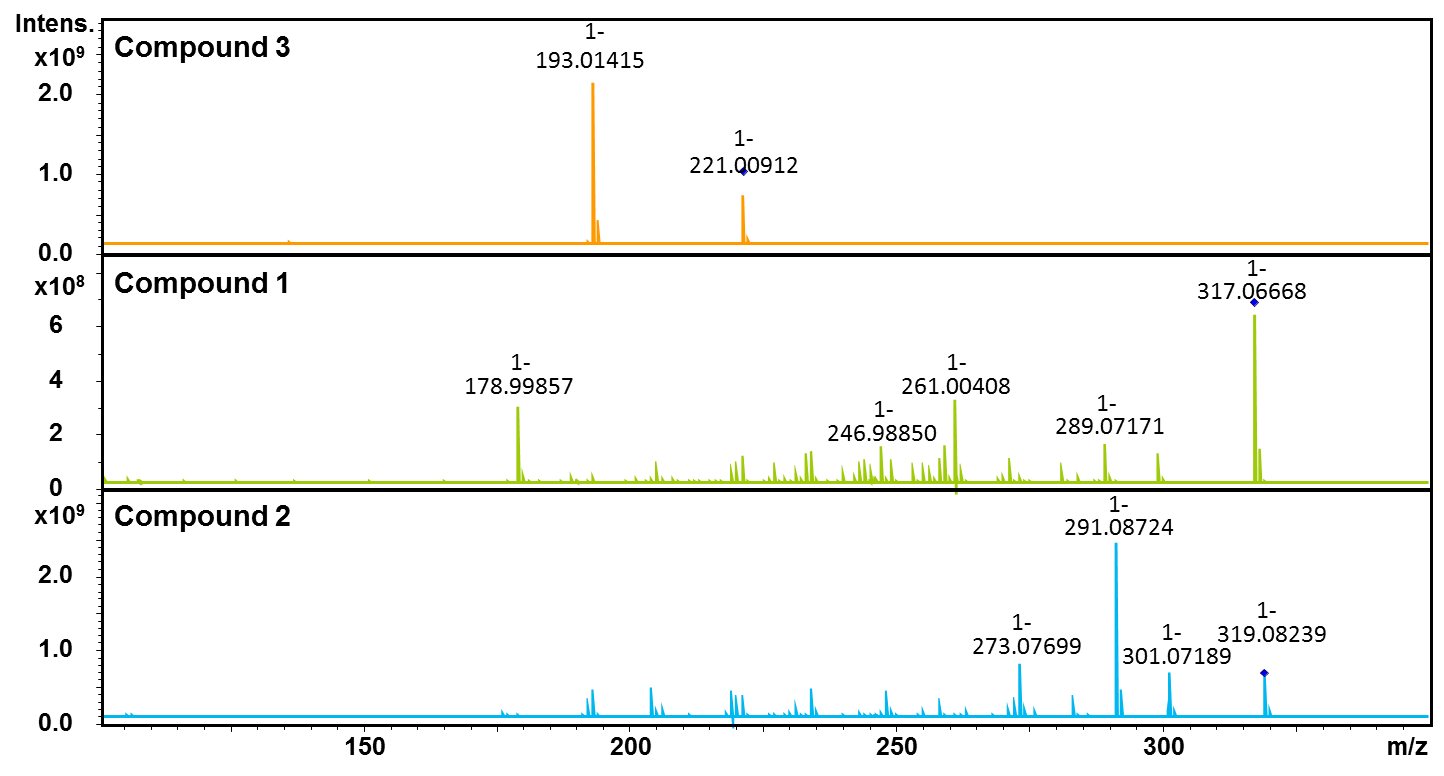


**Figure S1:** Collision-induced dissociation of quambalarine A (compound 1), quambalarine B (compound 2)
and mompain (compound 3).
