# Supplementary material for: Biologically Active Metabolites Produced by the Basidiomycete Quambalaria cyanescens
Source: PLoS One. 2015 Feb 27;10(2):e0118913. doi: 10.1371/journal.pone.0118913 (PMC4344228; doi:10.1371/journal.pone.0118913)
Supplement: S2 Fig — (PDF) [file pone.0118913.s002.pdf]

Supplementary material
Stodůlková et al. Biologically active metabolites produced by the basidiomycete *Quambalaria cyanescens*

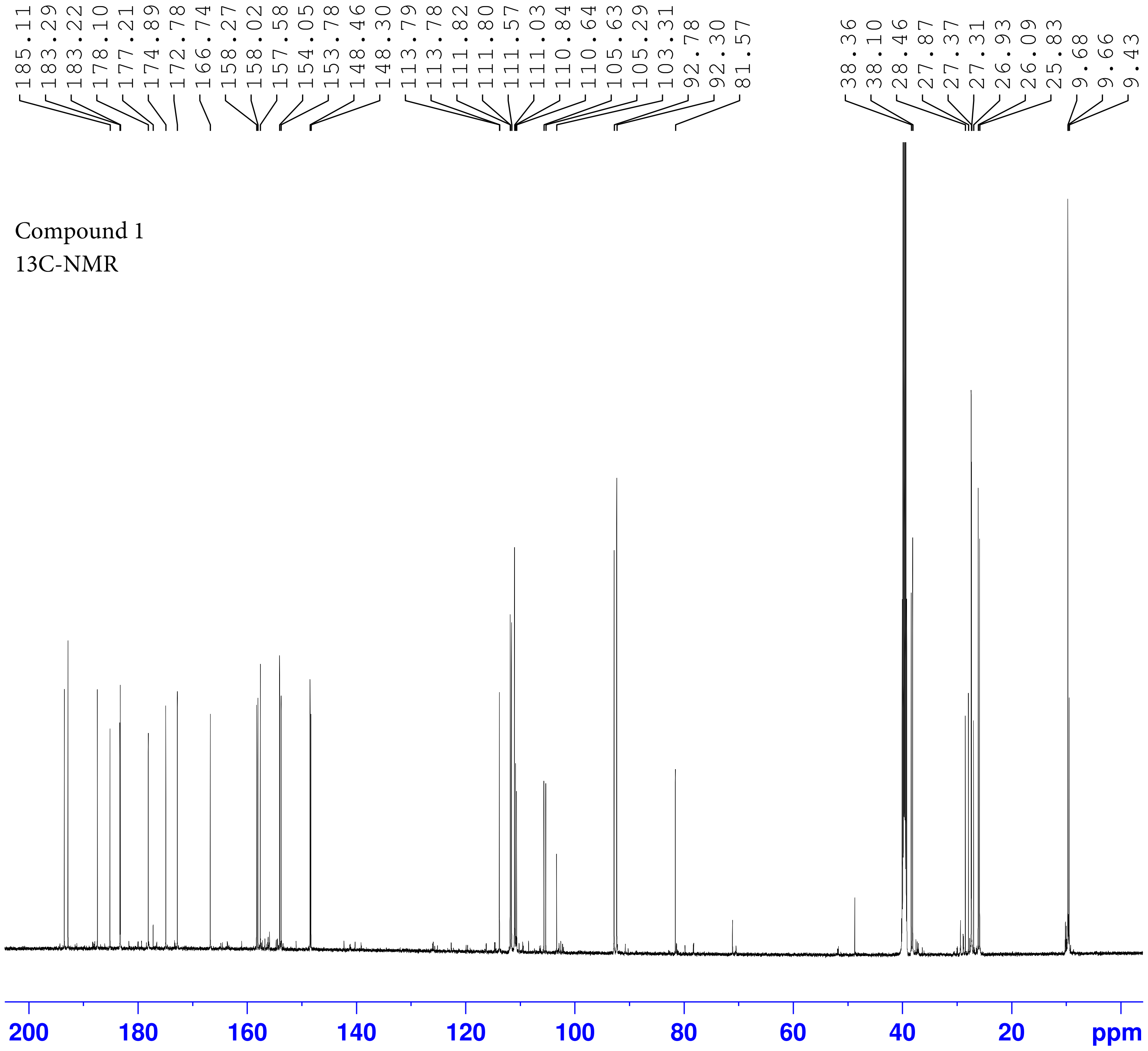


```
Current Data Parameters
NAME      FUGO-SK-2ndcryst
EXPNO                    2
PROCNO                   2

F2 - Acquisition Parameters
Date_             20090303
Time                 16.25
INSTRUM              spect
PROBHD   5 mm CPTCI 1H-
PULPROG               zgdc
TD                   65356
SOLVENT               DMSO
NS                    2395
DS                       4
SWH             36057.691 Hz
FIDRES           0.551712 Hz
AQ              0.9062698 sec
RG                    2050
DW                  13.867 usec
DE                  100.00 usec
TE                   303.2 K
D1             2.00000000 sec
D11            0.03000000 sec
TD0                      1

======== CHANNEL f1 ========
NUC1                   13C
P1                   12.00 usec
PL1                   0.30 dB
PL1W         107.13125610 W
SFO1          150.9430463 MHz

======== CHANNEL f2 ========
CPDPRG[2           waltz16
NUC2                    1H
PCPD2                80.00 usec
PL2                   4.70 dB
PL12                 24.70 dB
PL2W            5.66006804 W
PL12W           0.05660067 W
SFO2           600.2324009 MHz

F2 - Processing parameters
SI                  131072
SF             150.9280111 MHz
WDW                     EM
SSB      0
LB                    1.00 Hz
GB       0
PC                    1.40
```

Compound 2 (quambalarine B)
13C-NMR

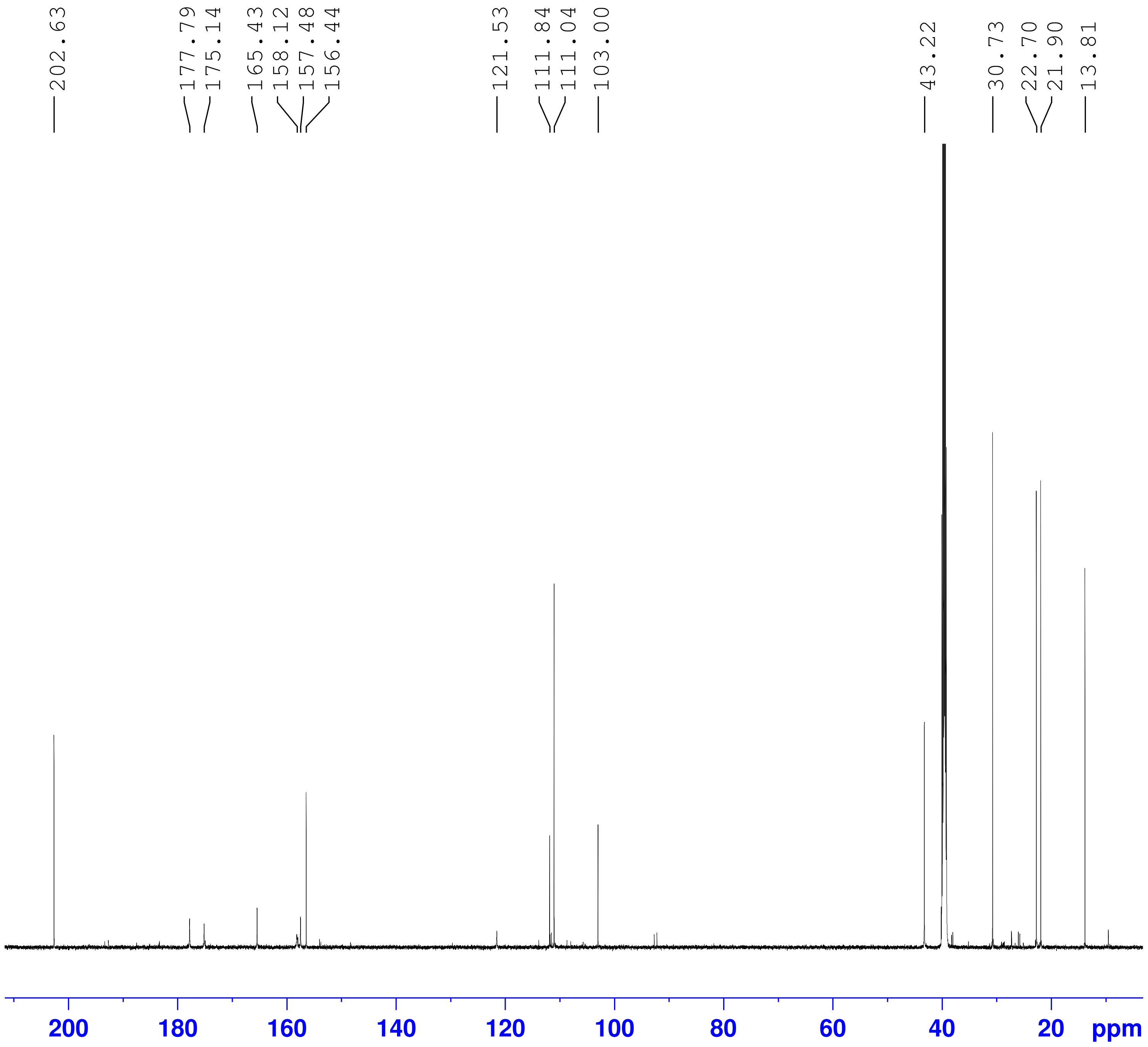


```
Current Data Parameters
NAME      Fialka-frakce5a
EXPNO                    2
PROCNO                   2

F2 - Acquisition Parameters
Date_             20120208
Time                  7.11
INSTRUM              spect
PROBHD    5 mm CPTCI 1H-
PULPROG               zgpg
TD                   65356
SOLVENT               DMSO
NS                    8192
DS                       4
SWH              36057.691 Hz
FIDRES            0.551712 Hz
AQ               0.9062698 sec
RG                    2050
DW                  13.867 usec
DE                   30.00 usec
TE                   303.1 K
D1              1.00000000 sec
D11             0.03000000 sec
L31                      1
TD0                      1

======== CHANNEL f1 ========
NUC1                   13C
P1                   12.00 usec
PL1                   0.30 dB
PL1W          107.13125610 W
SFO1           150.9430463 MHz

======== CHANNEL f2 ========
CPDPRG[2 bi_waltz65_256
NUC2                    1H
PCPD2                80.00 usec
PL2                   4.70 dB
PL12                 24.70 dB
PL13                 24.70 dB
PL2W            5.66006804 W
PL12W           0.05660067 W
PL13W           0.05660067 W
SFO2           600.2324009 MHz

F2 - Processing parameters
SI                  131072
SF             150.9280245 MHz
WDW                     EM
SSB       0
LB                    1.00 Hz
GB        0
PC                    1.40
```

Compound 1 (quambalarine A)
1H-NMR

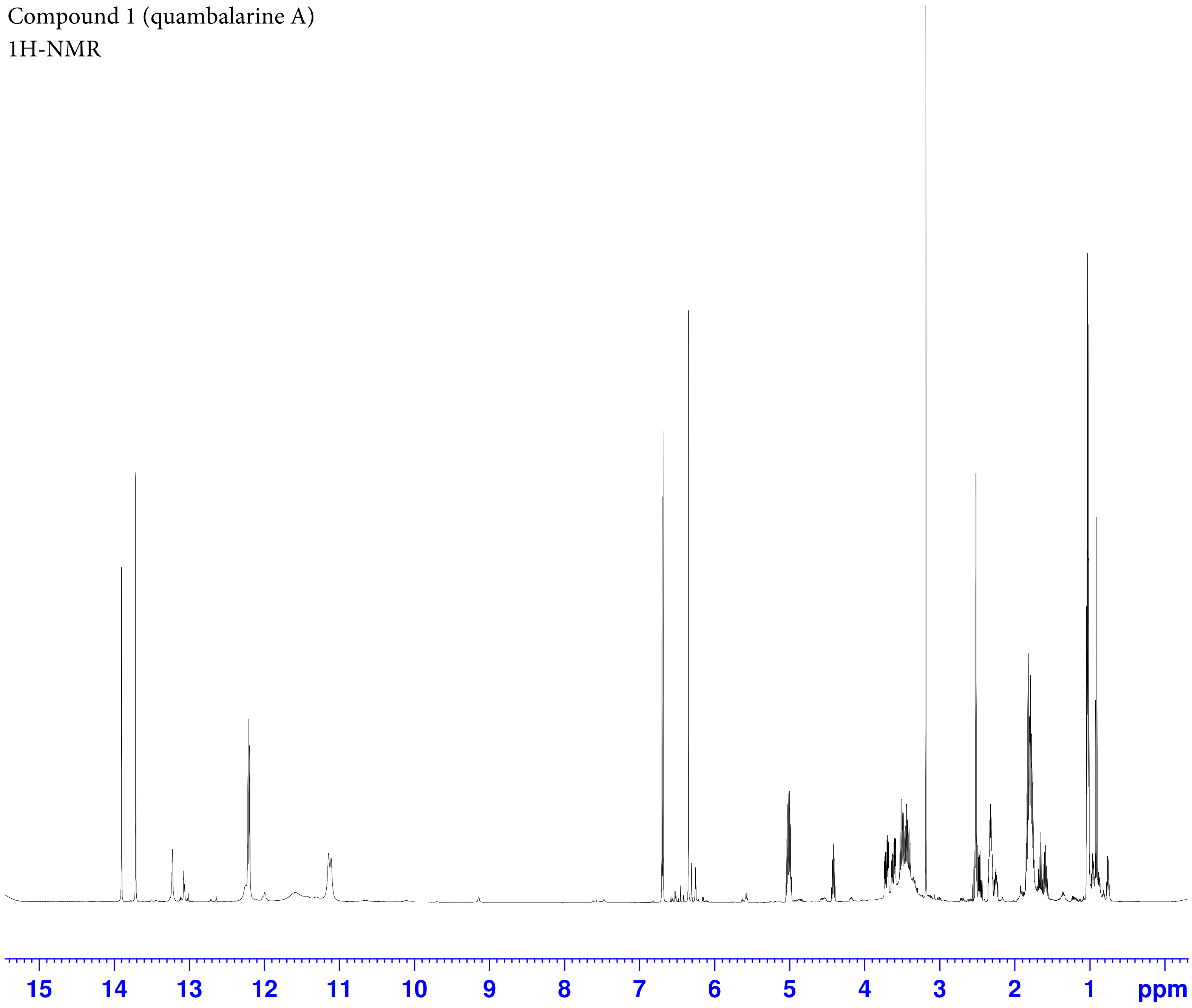

Current Data Parameters

NAME	FUGO-SK-2ndcryst
EXPNO	1
PROCNO	1

F2 - Acquisition Parameters

Date_	20090304
Time	14.45
INSTRUM	spect
PROBHD	5 mm CPTCI 1H-
PULPROG	zg30
TD	65536
SOLVENT	DMSO
NS	64
DS	4
SWH	9469.697 Hz
FIDRES	0.144496 Hz
AQ	3.4603007 sec
RG	40.3
DW	52.800 usec
DE	30.00 usec
TE	303.2 K
D1	1.00000000 sec
TD0	1

======== CHANNEL f1 ========

NUC1	1H
P1	8.00 usec
PL1	4.70 dB
PL1W	5.66006804 W
SFO1	600.2345443 MHz

F2 - Processing parameters

SI	32768
SF	600.2300000 MHz
WDW	EM
SSB	0
LB	0 Hz
GB	0
PC	1.40

Compound 2 (quambalarine B)
1H-NMR

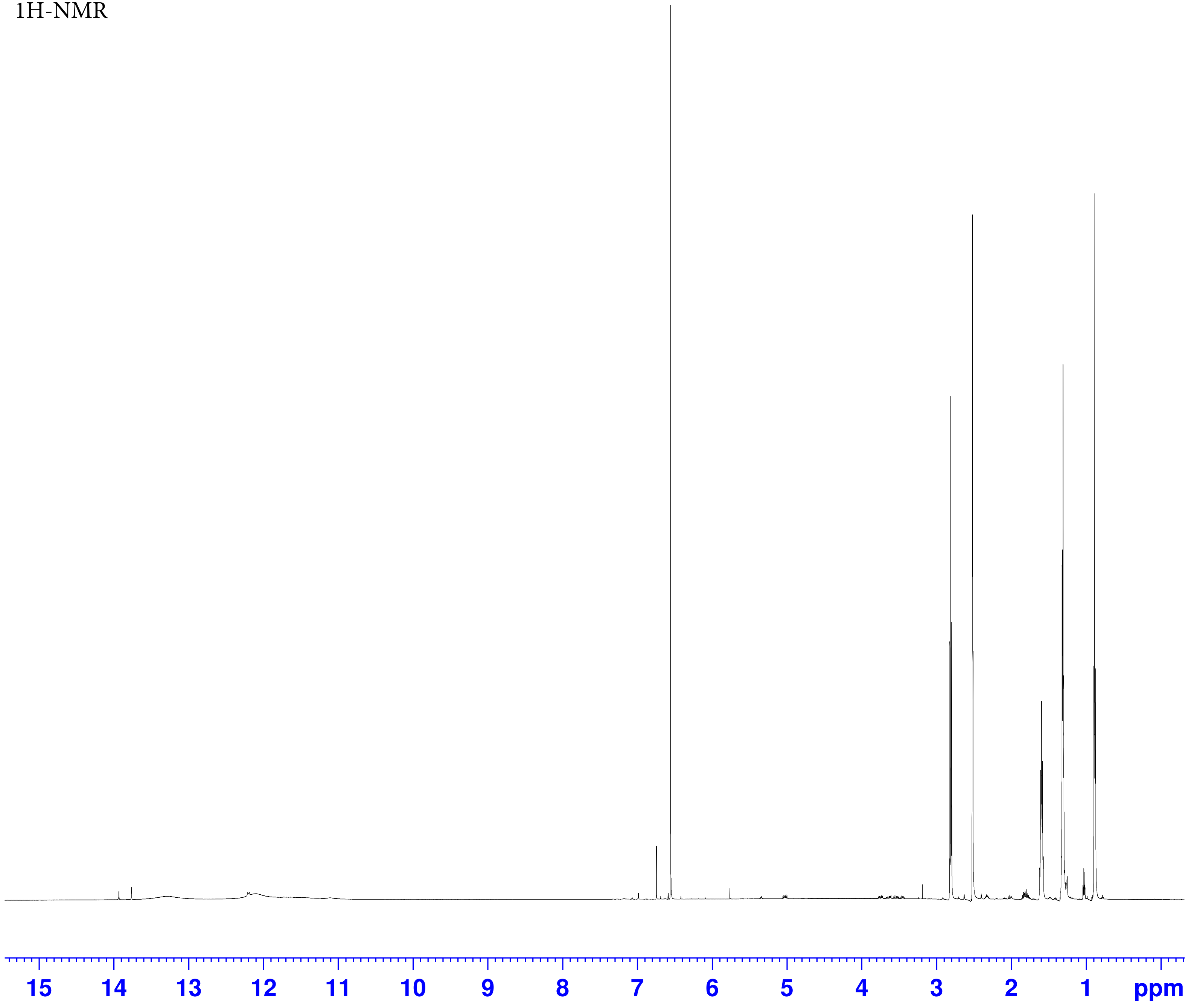

Current Data Parameters
NAME Fialka-frakce5a
EXPNO 1
PROCNO 1

F2 - Acquisition Parameters
Date_ 20120208
Time 6.36
INSTRUM spect
PROBHD 5 mm CPTCI 1H-
PULPROG zg
TD 131072
SOLVENT DMSO
NS 128
DS 4
SWH 11029.412 Hz
FIDRES 0.084148 Hz
AQ 5.9419308 sec
RG 8
DW 45.333 usec
DE 6.50 usec
TE 303.1 K
D1 1.00000000 sec
TD0 1

======== CHANNEL f1 ========
NUC1 1H
P1 8.00 usec
PL1 4.70 dB
PL1W 5.66006804 W
SFO1 600.2347826 MHz

F2 - Processing parameters
SI 262144
SF 600.2300000 MHz
WDW EM
SSB 0
LB 0 Hz
GB 0
PC 1.00
