# Supplementary material for: Biologically Active Metabolites Produced by the Basidiomycete Quambalaria cyanescens
Source: PLoS One. 2015 Feb 27;10(2):e0118913. doi: 10.1371/journal.pone.0118913 (PMC4344228; doi:10.1371/journal.pone.0118913)
Supplement: S3 Fig — (DOCX) [file pone.0118913.s003.docx]

Antibacterial and antifungal activity of naphthoquinones isolated from submerged culture of *Quambalaria cyanescens* and their comparison with commercial antibiotics. 20 μl (1 mg/mL) was loaded in all cases except of *A. fumigatus* and *C. albicans* where 50 μl (1 mg/mL) of crude extract, quambalarine A and B and mompain was used. Activities are expressed as a semi diameter of the observed growth inhibition zone (mm).

**Abbreviations used:** M – mompain; Sk – quambalarine A; F – quambalarine B; 936 – crude MeOH extract of *Q. cyanescens* CCF 3528; 1710 - crude MeOH extract of *Q. cyanescens* CCM 8372; 755 - crude MeOH extract of *Q. cyanescens* MK755; CH chloramphenicol; C – cycloheximide; S – Streptomycine; A –Antimycine A; MeOH – negative control.

| 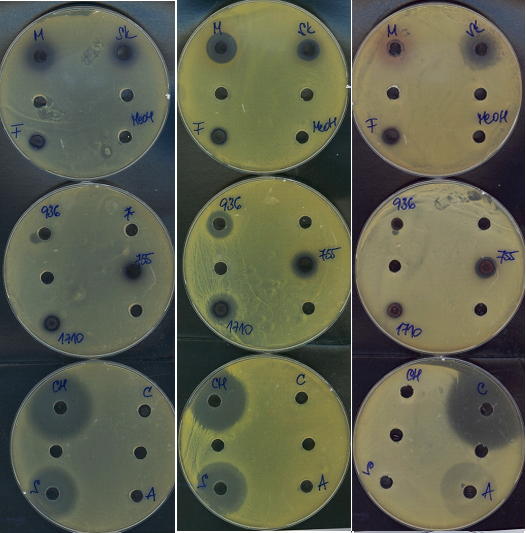 |
| --- |
| *Escherichia coli* ATCC 3988 *Kocuria rhizophila CCM 552 Saccharomyces cerevisiae* CCM 8191 |
|  |
| 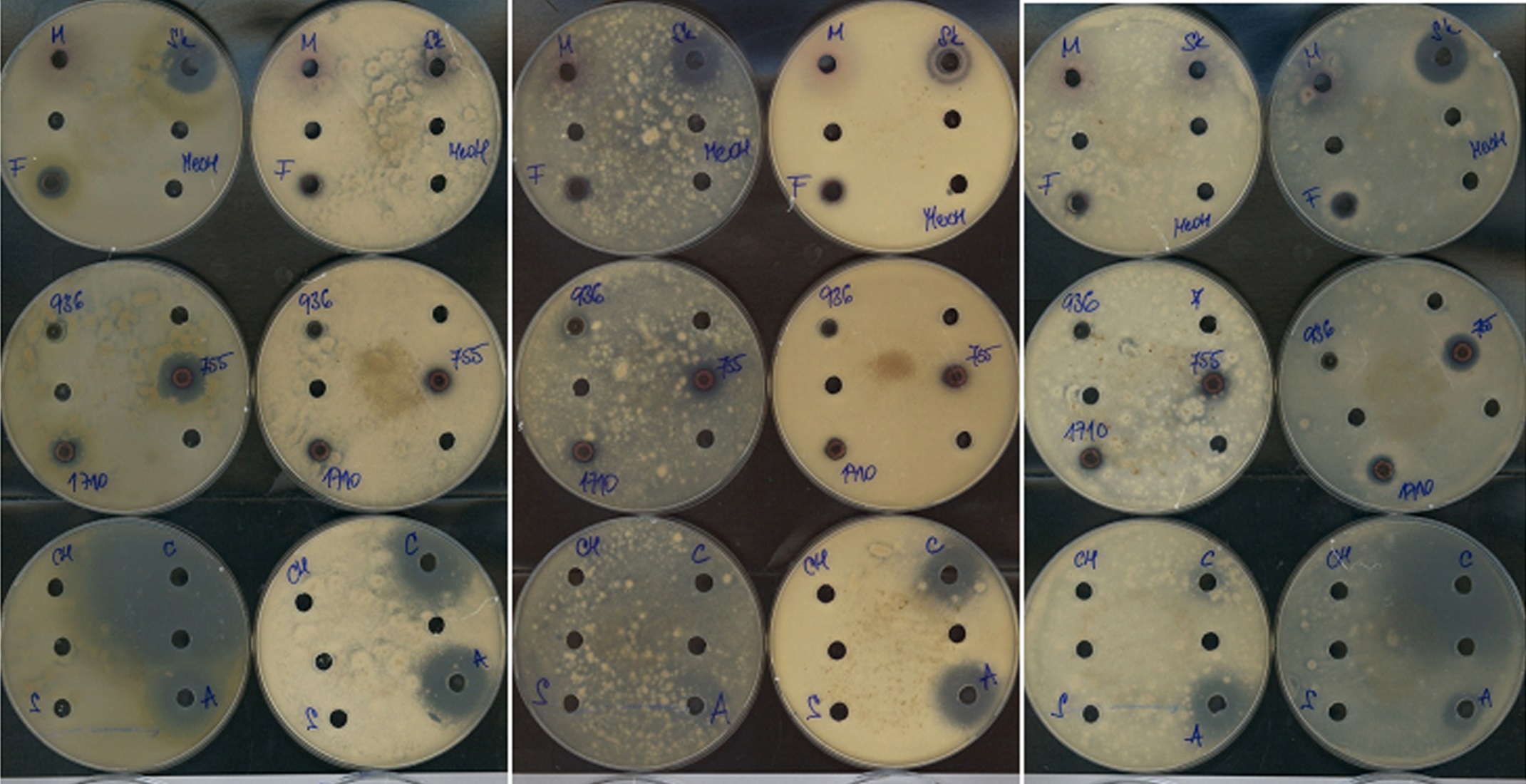 |
| *Geosmithia* sp. 9 strain RJ0258 *G. langdonii* CCF 3332 *Beauveria bassiana* strain CCF 4422 *Geosmithia* sp. 2 strain CCF 4273 *Penicillium decumbens* CCF 4423 *Graphium fimbriisporum* CCF 4421 |

| 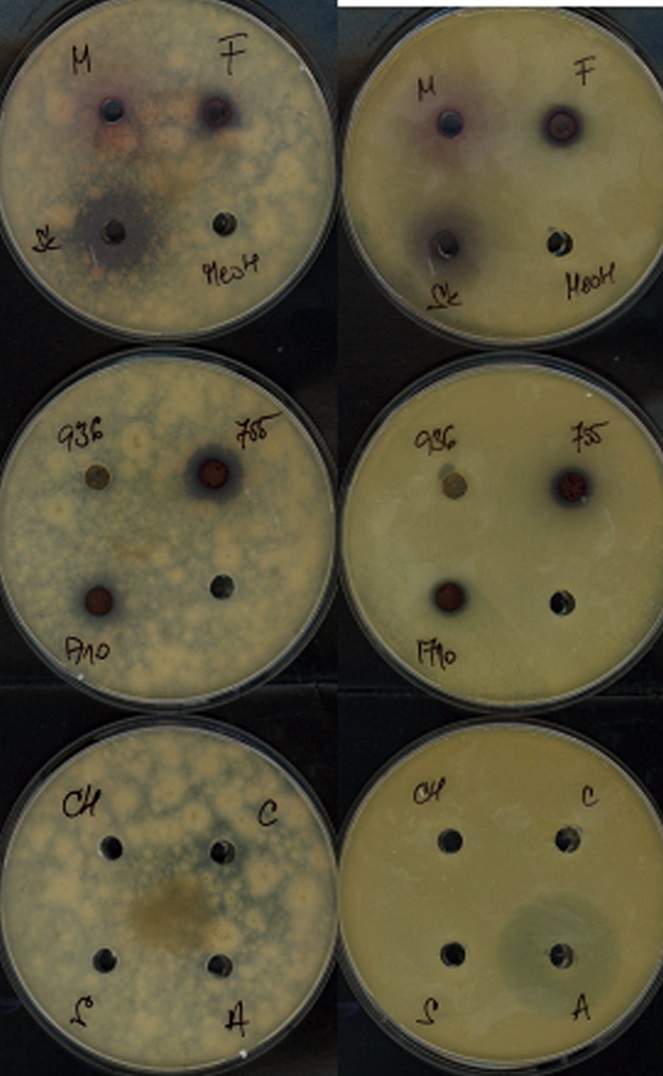 |
| --- |
| *Aspergillus fumigatus CEA10 Candida albicans 8558* |
