# Supplementary material for: Biologically Active Metabolites Produced by the Basidiomycete Quambalaria cyanescens
Source: PLoS One. 2015 Feb 27;10(2):e0118913. doi: 10.1371/journal.pone.0118913 (PMC4344228; doi:10.1371/journal.pone.0118913)
Supplement: S1 File — (DOCX) [file pone.0118913.s004.docx]

Effect of quambalarine B on Jurkat cell line - Quantitation of the intracellular MitoTracker® Red CMXRos signal

Biological activity of all isolated naphthoquinones was tested on suspension tumor-derived cell lines (REH, NALM 6 and Jurkat) using flow cytometry. Representative example of the FACS-based bioassay of the mitochondrial activity performed on Jurkat cell line is shown.

**Experimental procedure**

Tested cell types were cultivated in 96 well plates (Nunc, **Thermo Fisher Scientific**, Waltham, MA, USA) and treated with compounds dissolved in the DMSO (stock solution 10 mM) for various times and concentrations and measured using FACS LSR II (Becton Dickinson) for MitoTracker® Red CMXRos (Molecular Probes, Invitrogen, Carlsbad, CA, USA) signal.


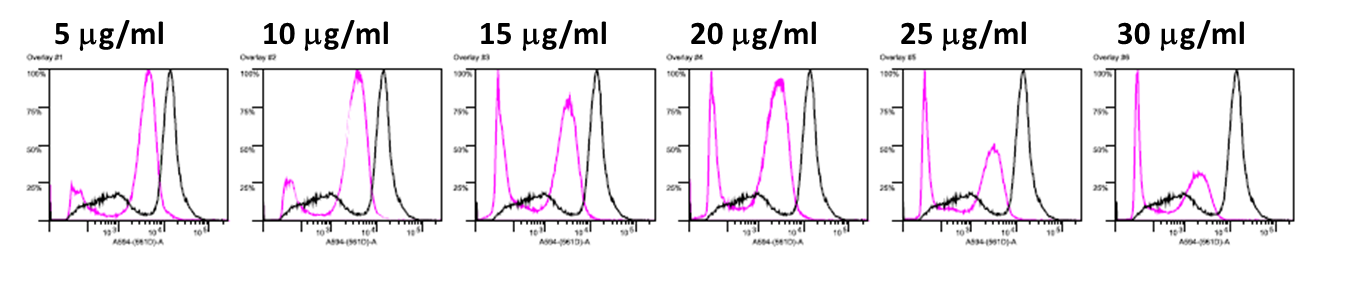


**Figure S3.** Quantitation of the MitoTracker® Red CMXRos signal after 24h treatment of Jurkat cell line with various concentrations of quambalarine B (5-30μg/ml). Fluorescence signal reflecting proton gradient presence in experimental conditions (red) is overlayed with the data acquired on the control cells (black line). Note the concentration-dependent drop in MitoTracker® Red CMXRos fluorescence intensity (logarithmic x-axis).
